# Predictive effect of the systemic inflammation response index (SIRI) on the efficacy and prognosis of neoadjuvant chemoradiotherapy in patients with locally advanced rectal cancer

**DOI:** 10.1186/s12893-024-02384-5

**Published:** 2024-03-13

**Authors:** Yuanyi Ding, Zining Liu, Jing Li, Wenbo Niu, Chenhui Li, Bin Yu

**Affiliations:** The Second General Surgery, Hebei Medical University Fourth Affiliated Hospital and Hebei Provincial Tumour Hospital, Hebei, 050011 China

**Keywords:** Systemic inflammation response index, Rectal cancer, Neoadjuvant chemoradiotherapy, Curative effect and prognosis

## Abstract

**Background:**

Inflammation is a part of tumours, and inflammatory cells can affect the proliferation, invasion, and development of tumour cells. An increasing number of peripheral blood inflammatory markers have been found to play very important roles in the treatment and prognosis of cancer patients. The systemic inflammatory response index (SIRI) is a newer inflammatory marker, and its role in colorectal cancer, especially in locally advanced rectal cancer, is still unclear.

**Methods:**

From 2015 to 2020, 198 patients with locally advanced rectal cancer (LARC) who underwent surgery following neoadjuvant chemoradiotherapy (Neo-CRT) were analysed. Patients were categorized into good- and poor- response groups according to their pathological results, and clinical characteristics and baseline parameters were compared between the two groups. The optimal cutoff values for inflammatory indicators were determined using receiver operating characteristic (ROC) analysis. Univariate and multivariate analyses were performed using the Cox proportional hazard model. Survival analysis was performed via the Kaplan‒Meier method.

**Results:**

After patients were grouped into good and poor response groups, indicator differences were found in CEA, neutrophil-to-lymphocyte ratio (NLR), systemic immune-inflammation index (SII), and SIRI. According to the ROC analysis, the NLR (*P* = 0.015), SII (*P* = 0.001), and SIRI (*P* = 0.029) were significant prognostic factors. After univariate and multivariate analyses of the Cox proportional hazards regression model, only the SIRI was found to be an independent prognostic factor for overall survival (OS) and disease-free survival (DFS). Finally, Kaplan‒Meier survival curves also confirmed the ability of the SIRI to predict survival.

**Conclusion:**

The preoperative SIRI can be used to predict the response to Neo-CRT in LARC patients and is an independent predictor of OS and DFS in postoperative patients. A high SIRI was associated with poor radiotherapy response and predicted poor OS and DFS.

## Introduction

As suggested by the International Agency for Research on Cancer, colorectal cancer (CRC) is the third most common malignant tumour and ranks second in mortality among all kinds of tumours, as suggested by the International Agency for Research on Cancer [1], of which rectal cancer accounts for 40% [2]. The diagnosis and treatment of rectal cancer may involve surgery, chemotherapy, radiotherapy, imaging evaluation, pathological evaluation, endoscopy and so on.

For locally advanced rectal cancer, neoadjuvant chemoradiotherapy (Neo-CRT) followed by total mesorectal excision can improve tumour staging and reduce the local recurrence rate [3]. However, the response to Neo-CRT varies among different patients. Studies have shown that patients who response well to Neo-CRT are less likely to suffer from local and distant recurrence [4] and have longer overall survival (OS) and disease-free survival (DFS) than patients who respond poorly [5].

Some studies have noted that inflammation occurs in the tumour microenvironment. Inflammatory cells may affect tumour cell proliferation, invasion, and development [6]. In recent years, studies have uncovered various blood-related inflammatory markers, such as the neutrophil-to-lymphocyte ratio (NLR) [7], platelet-to-lymphocyte ratio (PLR) [8], lymphocyte-to-monocyte ratio (LMR), systemic immune-inflammation index (SII) [9], prognostic nutritional index (PNI) [10], and systemic inflammation response index (SIRI). Their advantages are that they are easy to collect, relatively inexpensive, and can be accurately measured by standardized methods. However, the results of these studies are inconsistent [11]. Therefore, our study evaluated the predictive value of 9 inflammatory markers for the efficacy and prognosis of Neo-CRT in patients with locally advanced rectal cancer (LARC) and revealed that the SIRI was the most meaningful indicator.

## Methods

### Patient selection

This study included 207 patients with LARC who received preoperative Neo-CRT and radical surgery at the Fourth Hospital of Hebei Medical University from April 2015 to August 2020. The inclusion criteria were as follows: (1) patients with locally advanced rectal cancer; (2) pathologically confirmed rectal adenocarcinoma; (3) preoperative MRI, CT, and other examinations for accurate staging; (4) preoperative Neo-CRT performed according stage. During radiotherapy, an oral 5-fluorouracil drug was to have been given to sensitize the patient, and radical surgery needed to have been performed 8–12 weeks after radiotherapy. The exclusion criteria included the following: (1) patients with distant metastases found before surgery; (2) patients with previous antitumour therapy; (3) patients with missing clinical data; and (4) patients who were lost to follow-up. In total, 198 patients were included. By collecting and analysing preoperative blood inflammatory indices, postoperative pathology, postoperative survival, and recurrence and metastasis time, we studied the relationships between inflammatory indices and the efficacy of Neo-CRT, including the prognosis of Neo-CRT treated patients.

### Inflammatory markers

The counts of whole blood leukocytes, neutrophils, lymphocytes, monocytes, platelets, fibrinogen, D-dimer, and albumin were recorded within 1 week before surgery. The levels of nine widely studied markers, NLR, PLR, LMR, fibrinogen (Fib), D-dimer (D-dimer), SII (platelet count × neutrophil count/lymphocyte count), Advanced Lung Cancer Inflammatory Index (ALI) (BMI × albumin/NLR), PNI (albumin + 5×lymphocyte count), and SIRI (neutrophil count × monocyte count/lymphocyte count), were calculated.

### Evaluation of treatment response

The response to neoadjuvant chemoradiation was assessed using the AJCC 8th edition tumour regression grade (TRG) scoring system. The TRGs were scored as follows: TRG0 (withdrawal completely), no tumour cells visible under a microscope; TRG1 (near total withdrawal), only single or small foci of tumour cells were observed under a microscope; TRG2 (partial withdrawal), significant regression but residual tumour with excess single or small foci of tumour cells; TRG3 (poor or no withdrawal), extensive residual tumour without significant regression. A large number of studies [12, 13] have shown that when evaluating the efficacy of Neo-CRT, patients with TRG scores of 0 and 1 can be regarded as the good response group, and patients with TRG scores of 2 and 3 can be regarded as the poor response group.

### Statistical analysis

All the statistical analyses were performed with SPSS software version 25.0 for Windows (SPSS Inc., Chicago, IL, USA). *P* < 0.05 was considered indicative of a statistically significant difference. Continuous variables were compared between the two groups using the t test, whereas categorical variables were compared using the Chi-square test or Fisher’s exact test. Receiver operating characteristic (ROC) curve analysis and the area under the curve (AUC) were used to assess the ability of each inflammatory marker to predict the Neo-CRT response. The Youden index was used to determine the optimal cutoff thresholds for subsequent analysis. Overall survival (OS) was defined as the time from the date of diagnosis to the date of death from any cause, and disease-free survival (DFS) was defined as the time from the date of diagnosis to the date on which recurrence, either local or distant, was first detected or to the date of death from any cause. Univariate and multivariate Cox regression analyses were performed to assess predictors of prognosis. Variables with *P* < 0.05 in the univariate analysis were examined by multivariate analysis. Survival curves were constructed using the Kaplan–Meier method, with the log-rank test applied to compare survival curves.

## Results

### Patient characteristics and baseline parameters

The basic clinical characteristics of the patients included sex, age, BMI, clinical T stage, clinical N stage, length of newly diagnosed tumour, distance from the lower border of the tumour to the anal verge and depth of newly diagnosed tumour, CEA, CA 199, CA 724, and other information. Patient characteristics are shown in Table 1. Of the 207 patients we included, 9 were excluded due to the presence of distant metastases or missing follow-up information. Of the remaining 198 patients, 138 were male and 60 were female. A total of 104 patients were under 60 years old, and 94 were over 60 years old. A total of 107 patients had a BMI ≤ 25, and 91 patients had a BMI > 25. According to the postoperative pathology TRG score, the patients were categorized into a good response group (65 patients) and a poor response group (133 patients). There were significant differences between the two groups in CEA (*P* = 0.018), the NLR (*P* = 0.017), the SII (*P* = 0.018), and the SIRI (*P* = 0.009).

**Table 1:** Clinical characteristics and haematological indices of 198 patients

Variables	All	Good response	Poor response	P value
Gender				*P* = 0.818
Female	60(30.3%)	19(29.2%)	41(30.8%)	
Male	138(69.7%)	46(70.8%)	92(69.2%)	
Age				*P* = 0.141
≤60 years	104(52.5%)	39(60.0%)	65(48.9%)	
>60 years	94(47.5%)	26(40.0%)	68(51.1%)	
BMI				*P* = 0.969
≤25	107(54%)	35(53.8%)	72(54.1%)	
>25	91(46%)	30(46.2%)	61(45.9%)	
Clinical T stage				*P* = 0.523
T2-3	131(66.2%)	45(69.2%)	86(64.7%)	
T4	67(33.8%)	20(30.8%)	47(35.3%)	
Clinical N stage				*P* = 0.092
N-	6(3%)	4(6.2%)	2(1.5%)	
N+	192(97%)	61(93.8%)	131(98.5%)	
Length of newly diagnosed tumour				*P* = 0.511
≤5 cm	100(50.5%)	35(53.8%)	65(48.9%)	
>5 cm	98(49.5%)	30(46.2)	68(51.1%)	
distance from the lower border of the tumour to the anal verge				*P* = 0.265
≤5 cm	59(29.8%)	16(24.6%)	43(32.3%)	
>5 cm	139(70.2%)	49(75.4%)	90(67.7%)	
CEA				*P* = 0.018
≤5 µg/L	98(49.5%)	40(61.5%)	58(43.6%)	
>5 µg/L	100(50.5%)	25(38.5%)	75(56.4%)	
CA199				*P* = 0.582
≤40U/L	173(87.4%)	58(89.2%)	115(86.5%)	
>40U/L	25(12.6%)	7(10.8%)	18(13.5%)	
CA724				*P* = 0.747
≤6U/L	152(76.8%)	49(75.4%)	103(77.4%)	
>6U/L	46(23.2%)	16(24.6%)	30(22.6%)	
NLR (median)	4.83 ± 3.40	4.00 ± 1.94	5.23 ± 3.87	*P* = 0.017
PLR (median)	284.06 ± 147.38	267.52 ± 159.25	292.14 ± 141.14	*P* = 0.292
LMR (median)	2.19 ± 1.04	2.25 ± 1.11	2.15 ± 1.01	*P* = 0.559
Fib (median)	3.30 ± 0.75	3.18 ± 0.72	3.37 ± 0.76	*P* = 0.090
D-dimer (median)	0.26 ± 0.37	0.25 ± 0.37	0.26 ± 0.37	*P* = 0.890
SII (median)	976.21 ± 970.43	743.44 ± 602.26	1089.97 ± 1090.82	*P* = 0.018
ALI (median)	279.76 ± 147.15	312.76 ± 153.46	263.64 ± 141.77	*P* = 0.032
PNI (median)	45.02 ± 5.28	45.52 ± 3.77	44.77 ± 5.87	*P* = 0.280
SIRI (median)	1.87 ± 1.46	1.49 ± 0.84	2.06 ± 1.66	*P* = 0.009

### Receiver operating characteristic curve analysis of inflammatory markers

**Fig. 1:**
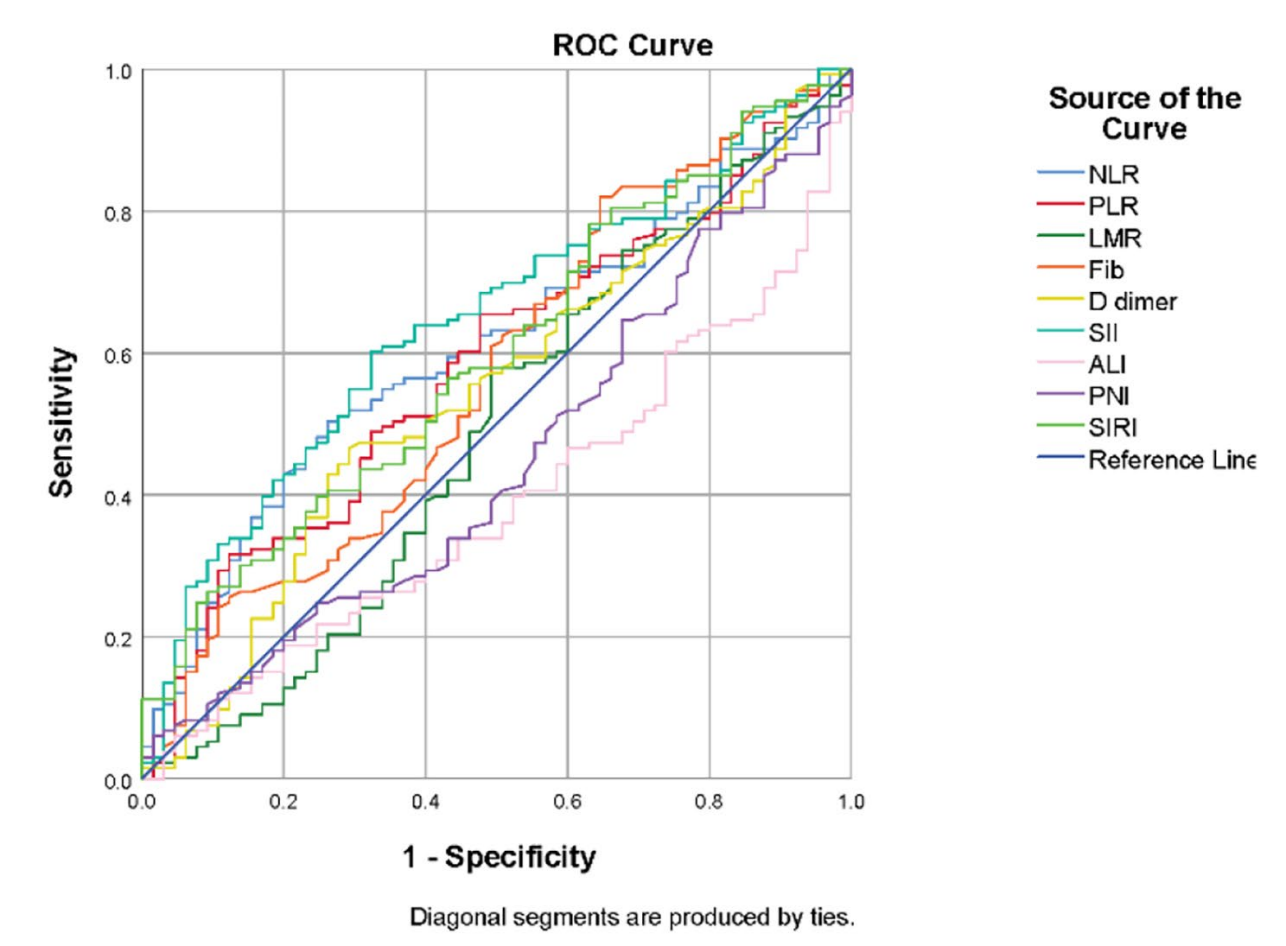
Receiver operating characteristic analysis of haematological parameters for the prediction of tumour response

ROC curve analysis was performed by dividing TRG scores 0–1 into clinical endpoints to determine which inflammatory markers were the best predictors of treatment response. We calculated 9 combinations of inflammatory markers to evaluate their predictive effects. Figure 1 shows the ROC curves of the 9 inflammatory markers. The AUC values ​​and P values ​​of the 9 inflammatory indicators are shown in Table 2. Among the indicators with an AUC ≥ 0.50, three had P values less than 0.05, namely, the NLR (*P* = 0.015), the SII (*P* = 0.001), and the SIRI (*P* = 0.029). Then, the optimal cutoff values of each indicator were calculated by selecting the largest Youden index. The optimal cutoff values ​​for the NLR, SII, and SIRI were 4.39, 707.65, and 2.53, respectively.

**Table 2:** AUC values from the ROC analysis of inflammatory markers for predicting the tumour response

	AUC	P value	95%CI	
			Lower bound	Upper bound
NLR	0.607	0.015	0.527	0.687
PLR	0.583	0.058	0.501	0.665
LMR	0.489	0.799	0.400	0.578
Fib	0.571	0.107	0.485	0.657
D-dimer	0.545	0.302	0.460	0.630
SII	0.645	0.001	0.567	0.724
ALI	0.389	0.011	0.310	0.469
PNI	0.449	0.246	0.365	0.534
SIRI	0.596	0.029	0.514	0.677

### Cox proportional hazards model and analysis of Kaplan‒Meier survival curves

The follow-up endpoint of this study was May 2022 or patient death. The median follow-up time was 42.8 months, of which the shortest follow-up time was 20.73 months, and the longest follow-up time was 89.83 months. According to the survival analysis, the median OS for all patients was 38.87 months. The median OS was 41.82 months for the 168 patients who did not die and 28.12 months for the 30 patients who died. Disease progression or death occurred in 61 patients at a median of 15.47 months, and the median DFS of the 137 patients who did not experience disease progression was 36.33 months.

Tables 3 and 4 show the Cox proportional hazard models established based on OS and DFS. In univariate analysis for OS, we found that a high SIRI, high CA 199 concentration (> 40 U/L) and deep newly diagnosed tumours were associated with worse OS. Multivariate analysis of the above three factors revealed, that high SIRIs and CA 199 levels were independent predictors of OS. Univariate analysis of DFS revealed that a high SIRI, high CA 724 (> 6 U/L), and deep newly diagnosed tumours were associated with poor DFS. However, after multivariate analysis, only a high SIRI was an independent poor prognostic factor for DFS. Therefore, we found that the preoperative SIRI was a predictor of OS and DFS in patients.

**Table 3:** Univariate and multivariate analysis of overall survival in 198 patients

Variables	Univariate, OR(95%CI)	P value	Multivariate, OR(95%CI)	P value
Gender		*P* = 0.697		
Female	1			
Male	0.856(0.391,1.873)			
Age		*P* = 0.186		
≤60 years	1			
>60 years	1.637(0.788,3.399)			
BMI		*P* = 0.156		
≤25	1			
>25	0.583(0.277,1.228)			
Clinical T stage		*P* = 0.429		
T2-3	1			
T4	1.343(0.646,2.791)			
Clinical N stage		*P* = 0.254		
-	1			
N+	0.434(0.103,1.824)			
Length of newly diagnosed tumour		*P* = 0.715		
≤5 cm	1			
>5 cm	1.035(0.838,1.278)			
Distance from the lower border of the tumour to the anal verge		*P* = 0.382		
≤5 cm	1			
>5 cm	1.459(0.626,3.402)			
Depth of newly diagnosed tumour	1.515(1.033,2.223)	*P* = 0.034	1.406(0.962,2.055)	*P* = 0.078
CEA		*P* = 0.377		
≤5 µg/L	1			
>5 µg/L	1.385(0.672,2.855)			
CA199		*P* = 0.001		*P* = 0.002
≤40U/L	1		1	
>40U/L	3.740(1.707,8.194)		3.563(1.624,7.814)	
CA724		*P* = 0.184		
≤6U/L	1			
>6U/L	1.674(0.783,3.578)			
NLR		*P* = 0.089		
≤4.39	1			
>4.39	1.873(0.910,3.856)			
SII		*P* = 0.092		
≤707.65	1			
>707.65	1.923(0.900,4.109)			
SIRI		*P* = 0.004		*P* = 0.009
≤2.53	1		1	
>2.53	2.870(1.393,5.910)		2.621(1.269,5.413)	

**Table 4:** Univariate and multivariate analysis of disease-free survival in 198 patients

Variables	Univariate, OR(95%CI)	P value	Multivariate, OR(95%CI)	P value
Gender		*P* = 0.824		
Female	1			
Male	1.066(0.609,1.868)			
Age		*P* = 0.709		
≤60 years	1			
>60 years	1.101(0.666,1.820)			
BMI		*P* = 0.156		
≤25	1			
>25	0.689(0.412,1.153)			
Clinical T stage		*P* = 0.458		
T2-3	1			
T4	1.218(0.724,2.049)			
Clinical N stage		*P* = 0.993		
-	1			
N+	1.007(0.246,4.124)			
Length of newly diagnosed tumour		*P* = 0.690		
≤5 cm	1			
>5 cm	1.108(0.670,1.833)			
Distance from the lower border of the tumour to the anal verge		*P* = 0.881		
≤5 cm	1			
>5 cm	1.043(0.601,1.809)			
Depth of newly diagnosed tumour	1.341(1.017,1.770)	*P* = 0.038	1.277(0.960,1.699)	*P* = 0.093
CEA		*P* = 0.836		
≤5 µg/L	1			
>5 µg/L	0.948(0.573,1.570)			
CA199		*P* = 0.227		
≤40U/L	1			
>40U/L	1.519(0.771,2.994)			
CA724		*P* = 0.033		*P* = 0.122
≤6U/L	1		1	
>6U/L	1.789(1.047,3.057)		1.539(0.891,2.658)	
NLR		*P* = 0.053		
≤4.39	1			
>4.39	1.641(0.993,2.714)			
SII		*P* = 0.051		
≤707.65	1			
>707.65	1.675(0.998,2.812)			
SIRI		*P* = 0.001		*P* = 0.005
≤2.53	1		1	
>2.53	2.381(1.411,4.018)		2.143(1.259,3.650)	

**Fig. 2:**
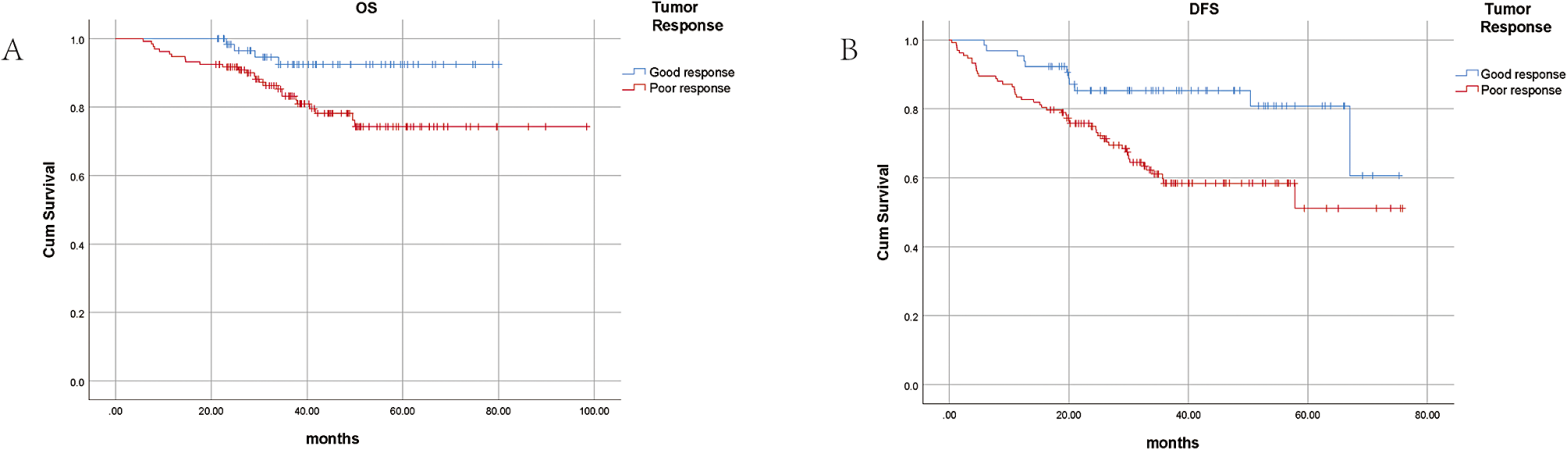
K-M curve of overall survival (**A**) and disease-free survival (**B**) between the good response group and poor response group

**Fig. 3:**
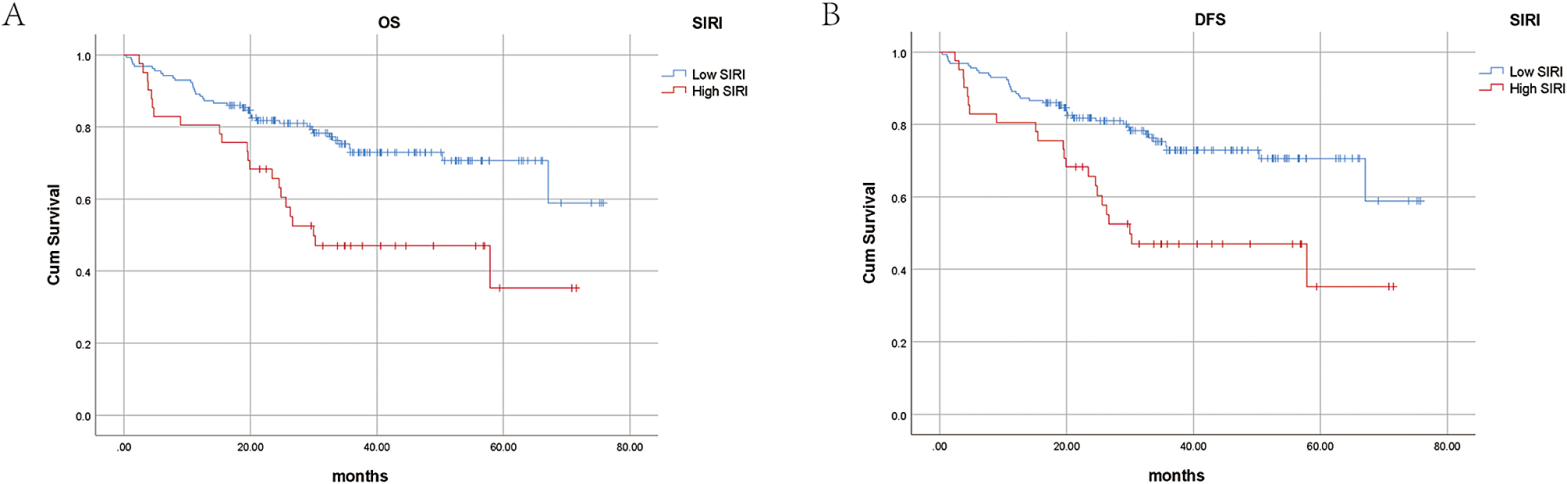
K-M curve of overall survival (**A**) and disease-free survival (**B**) between the low SIRI group and high SIRI group

The results are shown in Figs. 2 and 3. The OS (*P* = 0.017) and DFS (*P* = 0.003) of the patients in the good response group were significantly better than those in the poor response group. According to the Kaplan‒Meier survival curve of the SIRI, the OS (*P* = 0.003) and DFS (*P* = 0.001) of the low SIRI group were significantly better than those of the high SIRI group. Therefore, patients in the low SIRI group and the good response group had a better prognosis.

## Discussion

Patients with locally advanced rectal cancer can benefit more from preoperative Neo-CRT combined with surgery than from other treatment methods. However, there are still no clear indicators for predicting the efficacy of neoadjuvant chemoradiotherapy or the prognosis of patients. In recent years, growing evidence has suggested that inflammation is associated with tumour progression and poor prognosis [14]. The systemic inflammatory response caused by tumours has a certain procancer effect, and the inflammatory mediators and cellular effectors produced by the inflammatory response can promote cell proliferation, induce angiogenesis, and increase genomic instability [15]. Therefore, the use of inflammatory markers to predict the prognosis of cancer patients may be a good strategy.

In recent years, many inflammatory markers, such as the NLR, SIRI, SII and other indicators, have been widely studied. Therefore, the discovery of highly sensitive inflammatory markers for evaluating treatment efficacy or predicting patient prognosis has become a clinically important issue. Chen et al. reported that a high NLR is an independent poor prognostic marker in colorectal cancer patients [16]. Moreover, a large number of studies have shown that a high NLR is a predictor of poor response after Neo-CRT and predicts poor OS and DFS in patients [17–19]. In addition, Chen et al. studied the data of 1383 colorectal cancer patients who underwent surgery and reported that the SII was a more effective predictor of postoperative survival in patients with colorectal cancer than were the NLR and PLR [20]. The SIRI was first proposed by Qi et al. in 2016 [21]. Studies have shown that the SIRI has greater prognostic value than other inflammatory markers in locally or locally advanced renal clear cell nephropathy [22], nasopharyngeal carcinoma [23], and esophagogastric junction cancer [24].

The systemic inflammatory response index (SIRI) is a novel inflammatory marker related to neutrophils, lymphocytes, and monocytes in the blood. Increased neutrophils can lead to the upregulation of neutrophil-associated chemokines [25], and various chemokines cause DNA damage [26] and gene mutations that induce tumour cell development [27]. Second, the cytokines and acidic environment produced by increased neutrophils can inhibit the anticancer activity of T cells and NK cells and promote tumour vascularization by releasing granulin and proangiogenic chemokines, causing metastasis [28]. Lymphocytes play a key role in tumour immunosuppression. Many studies have proven that lymphocytes play a beneficial role in tumour immunity by eliminating tumour cells, and a reduction in lymphocytes often indicates a poor prognosis [29]. Monocytes also play important roles in tumorigenesis and metastasis. In the tumour microenvironment, monocytes can differentiate into tumour-associated macrophages (TAMs), inhibit the adaptive immune response, and participate in tumour growth and metastasis. Higher monocyte levels may indicate poorer prognosis [30]. Therefore, the SIRI, as an index representing three cell counts, can play a predictive role in the poor prognosis of patients with malignant tumours [31].

Regarding pre-treatment SIRI values, high SIRI was found in a study to be associated with poor clinical outcomes [32]. Dong et al. reported that breast cancer patients who received neoadjuvant chemotherapy had a lower pretreatment SIRI and were more likely to achieve pCR, improving the success rate of treatment [33]. Hu et al. repored that the SIRI in peripheral blood before treatment was an independent predictor of OS in patients with non-small cell lung cancer receiving chemoradiotherapy, and for patients with different TNM stages, a low SIRI was a protective factor for prognosis [34]. In addition, there was a correlation between the SIRI and the response to chemotherapy. In patients with metastatic pancreatic cancer treated with mFOLFIRINOX, an increase in the SIRI (> 2.3) predicted a greater benefit from treatment [35]. A large number of studies have shown that the SIRI has a certain predictive effect on the prognosis of patients with gastric cancer, head and neck squamous cell carcinoma, renal cell carcinoma, ovarian cancer, and other tumours [36–39]. However, there are few studies on the SIRI in rectal cancer patients receiving neoadjuvant chemoradiotherapy.

Our study showed that the SIRI was clear predictive regarding the efficacy and prognosis of neoadjuvant chemoradiotherapy in patients with rectal cancer. First, we found differences in CEA, the NLR, the SII, and the SIRI after stratification by good or poor responses. Second, according to the ROC curve, indicators with an AUC > 0.5, for which the *P* values ​​of the NLR, SII, and SIRI were less than 0.05, were selected and the best cutoff values ​​of these three indicators were calculated for grouping. After univariate and multivariate analyses in the Cox proportional hazards regression model, the SIRI was found to be an independent predictor of OS and DFS in patients. After Kaplan‒Meier curve analysis, we found that the OS (*P* = 0.017) and DFS (*P* = 0.003) in the good response group were significantly better than those in the poor response group, and the OS (*P* = 0.003) and DFS (*P* = 0.001) of patients in the low SIRI group were also significantly better than those of the high SIRI group. In patients with higher SIRIs (> 2.53), there was poor responsiveness to neoadjuvant chemoradiotherapy and a poor survival prognosis. To benefit patients these patients, we should provide better treatment plans and review changes in condition more closely.

This was a single-centre study with a small sample size. Multicentre, large-sample research is needed in the future. Second, this was a retrospective study, and some comorbidities may have affected the patient’s haematological indices, thereby influencing the results. In addition, there are large differences in the optimal diagnostic cutoff for SIRI used by different studies. The optimal cutoff value in our study was 2.53, but further research is needed for verification.

## Conclusion

The preoperative systemic inflammatory response index (SIRI) predicts the response to Neo-CRT in patients with locally advanced rectal cancer and is an independent predictor of patient OS and DFS. A high SIRI is often associated with poor Neo-CRT response and predicts poor OS and DFS. Therefore, preoperative assessment of the SIRI in patients has a certain guiding importance.

## Data Availability

The datasets used and/or analysed during the current study are available from the corresponding author upon reasonable request.

## Abbreviations

SIRI: Systemic inflammation response index
Neo-CRT: Neoadjuvant chemoradiotherapy
LARC: Locally advanced rectal cancer
ROC: Receiver operating characteristic
NLR: Neutrophil-to-lymphocyte ratio
SII: Systemic immune-inflammation index
OS: Overall survival
DFS: Disease-free survival
CRC: Colorectal cancer
PLR: Platelet-to-lymphocyte ratio
LMR: Lymphocyte-to-monocyte ratio
PNI: Prognostic nutritional index
Fib: Fibrinogen
D-dimer: D-dimer
ALI: Advanced Lung Cancer Inflammatory Index
TRG: Tumour regression grade
AUC: Area under the curve
TAMs: Tumour-associated macrophages
